# Pre-procedural and intra-procedural computerized tomography: providing a roadmap for successful adrenal venous sampling procedures

**DOI:** 10.1007/s00261-024-04321-9

**Published:** 2024-05-13

**Authors:** Darius Jonasch, Peiman Habibollahi, A. Kyle Jones, Rony Avritscher, Mouhammed Amir Habra, Nancy D. Perrier, Paul H. Graham, Steven Y. Huang

**Affiliations:** 1 Division of Diagnostic Imaging, Department of Interventional Radiology, The University of Texas MD Anderson Cancer Center, 1400 Pressler St, Houston, TX 77030 USA; 2 Division of Diagnostic Imaging, Department of Imaging Physics, The University of Texas MD Anderson Cancer Center, 1400 Pressler St, Houston, TX 77030 USA; 3 Division of Internal Medicine, Department of Endocrine Neoplasia and Hormonal Disorders, The University of Texas MD Anderson Cancer Center, 1400 Pressler St, Houston, TX 77030 USA; 4 Division of Surgery, Department of Surgical Oncology, The University of Texas MD Anderson Cancer Center, 1400 Pressler St, Houston, TX 77030 USA

**Keywords:** Adrenal vein sampling, Pre-procedural intra-procedural computed tomography

## Abstract

**Background and purpose:**

Adrenal venous sampling (AVS) is used for the diagnosis of primary hyperaldosteronism. Technical difficulties with right adrenal vein (RAV) catheterization can lead to erroneous results. Our purpose was to delineate the location of the RAV on pre-procedural CT imaging in relation to the location identified during AVS and to report on the impact of successful RAV cannulation with and without the use of intra-procedural CT scanning.

**Methods:**

Retrospective case series including patients who underwent AVS from October 2000 to September 2022. Clinical and laboratory values were abstracted from the electronic medical record. Successful cannulation of the RAV was defined as a selectivity index > 3.

**Results:**

110 patients underwent 124 AVS procedures. Pre-AVS CT imaging was available for 118 AVS procedures. The RAV was identified in 61 (51.7%) CT datasets. Biochemical confirmation of successful RAV cannulation occurred in 98 (79.0%) of 124 AVS procedures. There were 52 (85.2%) procedures in which the RAV was identified on pre-AVS CT and there was biochemical confirmation of successful RAV sampling. Among these 52 procedures, the RAV was localized during AVS at the same anatomic level or within 1 vertebral body level cranial to the level identified on pre-AVS CT in 98.1% of cases. The rate of successful RAV cannulation was higher in patients who underwent intra-procedural CT (93.8% versus 63.9%), *P* < 0.01.

**Conclusions:**

Pre-AVS and intra-procedural CT images provide an invaluable roadmap that resulted in a higher rate of accurate identification of the RAV and successful AVS procedures; in particular, search for the RAV orifice during AVS can be limited to 1 vertebral body cranial to the level identified on pre-AVS CT imaging and successful cannulation can be confidently verified with intra-procedural CT.

## Introduction

Primary aldosteronism (PA) is a frequent cause of secondary hypertension. PA is increasingly seen as underdiagnosed [1, 2]. There is a spectrum of presentations in patients with PA ranging from asymptomatic cases to florid cases of uncontrolled hypertension that can be poorly responsive to multiple antihypertensive medications and therapies causing end organ damage [3]. Most common causes of PA include aldosterone-secreting adrenal adenoma and bilateral adrenal hyperplasia.

Adrenal venous sampling (AVS) has become the gold standard in the diagnosis of PA to differentiate unilateral vs. bilateral aldosterone overproduction in patients older than 35 years old [4]. AVS is a procedure by which blood is acquired directly from the right adrenal vein (RAV), left adrenal vein (LAV), and a peripheral vein. The right and left adrenal veins contain higher concentrations of adrenal hormones (i.e., aldosterone and cortisol) relative to the peripheral blood. Therefore, direct sampling of adrenal venous blood can confirm a functioning unilateral adrenal adenoma and bilateral adrenal hyperplasia. Discordance between an imaging identification of an adrenal nodule and localization of aldosterone hypersecretion via AVS can be present in up to 20% of cases, underscoring the clinical importance of AVS [5–7]. Specific lateralization ensures that the correct side has been identified for resection. If not performed properly and the results are inadequate, the consequences can be devastating by resulting in the wrong gland removed, no gland removed or hematoma and injury to the vein that decrease functional capacity of the adrenal.

Despite the clinical utility of AVS, the procedure can be technically challenging with first time success rates as low as 70% [8–11]. The low success rates are attributed to difficulty with RAV catheterization, which may result from a host of factors [8]. Accurate identification of the RAV is dependent on operator experience, as it has numerous anatomic appearances. Confusion with an accessory hepatic vein is also possible. Anatomically, the RAV is short, 2–3 mm in diameter, and arises either directly from the inferior vena cava (IVC) or an accessory hepatic vein [8]. In contrast to catheterization of the RAV, successful catheterization of the LAV is performed with nearly 100% accuracy given that the LAV and phrenic vein confluence arises directly from the cephalad aspect of the left renal vein.

CT imaging may provide invaluable anatomic information that can improve successful RAV catheterization [12]. The location of the RAV can be inferred from a preprocedural CT [13–16]. In a study by Omura K et al. the authors correlated RAV orifice location on pre-AVS CT imaging with RAV orifice location during AVS and found that 84% of RAVs localized during AVS to within 1 vertebral body cranial or caudal to the location seen on CT [14]. Another technique that offers the potential to improve successful RAV catheterization is intraprocedural CT [17–21]. In a meta-analysis of 809 patients, Hefezi-Nejad et al. demonstrated that success rates of RAV catheterization improved 19.8% (*P* < 0.0001) with the addition of intraprocedural CT [21]. The purpose of this study was two-fold. First, we sought to evaluate the location of the RAV orifice on pre-AVS CT imaging relative to the location of the RAV during AVS to determine if the location of the RAV could be further refined based on pre-AVS CT imaging. Second, we evaluated the effect upon successful RAV catheterization in our own cohort of patients who underwent intraprocedural CT during AVS compared to a cohort of patients who did not undergo intraprocedural CT during AVS.

## Methods

This retrospective cohort study was IRB-approved with waiver of informed consent granted. Patients with an AVS procedure at our institution between October 2000 and September 2022 were included in this study. Patient demographics, laboratory values (aldosterone and cortisol), radiographic images, and procedural details were abstracted from the electronic health system. Pre-AVS CT images during the arterial and venous phases of imaging (if available) and intraprocedural fluoroscopic images and CT images (if acquired) were evaluated.

**Fig. 1:**
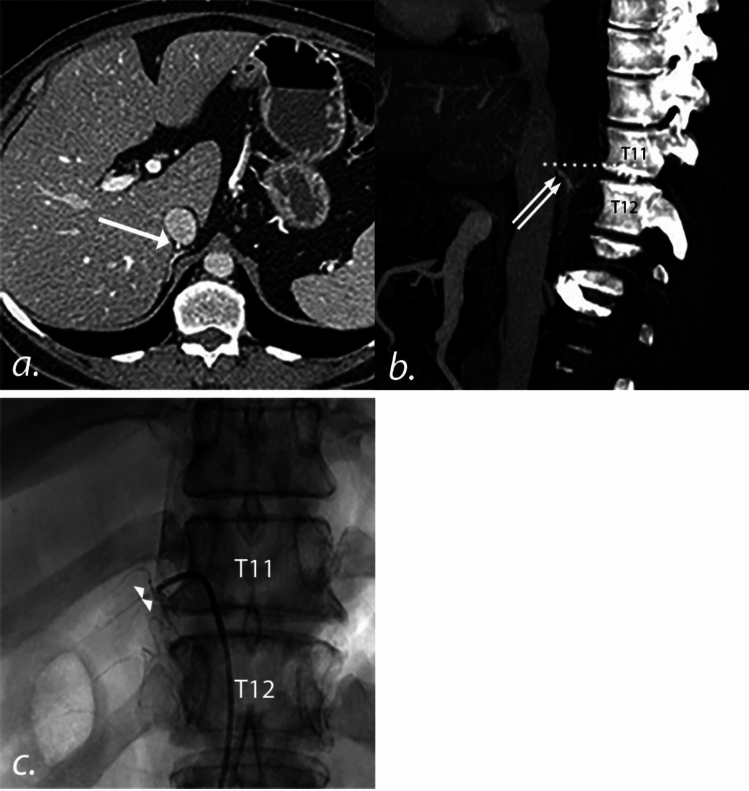
CT imaging and intraprocedural fluoroscopic images during AVS. **a** Axial CT images from a pre-AVS CT scan demonstrates the origin of the RAV (*arrow*). **b** Sagittal reformatted CT image from the pre-AVS CT scan demonstrates that the RAV origin (*double arrow*) maps to the lower third of the T11 vertebral body (dotted line). **c** Intraprocedural fluoroscopic image during AVS demonstrates the RAV (as confirmed with a SI > 3, *double arrowhead*) at the level of the lower third of the T11 vertebral body. *AVS* Adrenal vein sampling, *RAV* right adrenal vein, *SI* selectivity index

Pre-AVS CT images were evaluated for all patients included in our study for identification of the RAV. The location of the RAV orifice relative to the spine was identified on axial images using the arterial phase (if acquired) and venous phases of imaging. An anatomic level assigned based on the closest 1/3rd of a vertebral body or intervertebral disk space. In a similar fashion, the location of RAV during AVS was recorded based on fluoroscopic imaging during mid-expiration (Fig. 1). The location of the RAV based on the pre-AVS CT images and fluoroscopic images from the AVS procedure were considered concordant if two criteria were met: (1) RAV identified in the pre-AVS CT scan and (2) selectivity index (SI, ratio of cortisol concentration in the respective adrenal vein to that of the peripheral vein) > 3 for the sample obtained from the RAV.

AVS procedures were performed by 1 of 3 interventional radiologists (PH, RA, and SH) with 10 to 20 years of experience performing the procedure. The authors utilized the sequential sampling technique and ensured that aliquots from the adrenal veins and peripheral vein were taken within 10 min of one another as prior studies have shown that adrenal activity is relatively stable within this time [22]. Patients were given cosyntropin intravenously at 50 µg per hour beginning 1 h prior to the procedure. Cosyntropin was continued throughout the procedure. Following right common femoral vein access, a 5-French introducer sheath (PINNACLE®, TERUMO Medical Corporation, Somerset, NJ) was placed. A small (approximately 2 mm) hole was cut along the upper surface of the distal tip of a 5-French C2, Mikaelsson, or Simmons 1 catheter (Cook Medical, Bloomington, IN). The catheter was then used to catheterize the RAV. At the discretion of the interventional radiologist, an intraprocedural CT may have been performed to confirm successful cannulation of the RAV. Depending on the equipment available in the procedure room during AVS, intraprocedural CT (*n* = 64) was acquired as either cone-beam CT (CBCT) (*n* = 27 [42.2%]; DynaCT on Artis Q or Artis zeego, Siemens Healthineers USA, Malvern, PA) or multidetector CT (MDCT) in a hybrid angio-CT room (*n* = 37 [57.8%], Definition Edge, Siemens Healthineers USA, Malvern, PA). CBCT was acquired using the full image receptor for a field of view (FOV) of approximately 24 × 24 × 17.9 cm at isocenter and a 210° rotation lasting approximately 6 s. Exposure parameters were set automatically by the automatic exposure control (AEC) logic within the default 6sDCT Body protocol. The reconstructed voxel size was an isotropic 0.47 mm^3^.Both of the patient’s arms were raised over the head during image acquisition and breath holds were requested from the patient. Intraprocedural MDCT was acquired using the interventional imaging mode of the Definition Edge (i-Spiral), with the following settings: 120 kV (CarekV on, Slider Position 9, reference kV of 120), quality reference mAs of 300, 0.5 s rotation time, pitch of 1.0, detector configuration of 32 × 1.2, 5 mm image thickness and Bf37 kernel, and a scan delay after contrast administration of 38 s. The MDCT scan range included 2 vertebral bodies superior and 2 vertebral bodies inferior to the catheter location within the RAV orifice based on the initial CT topogram. Intraprocedural CBCT scans were acquired during mid-expiration and the arms were raised above the patient’s head. Intraprocedural MDCT scans were acquired without breath holding and the arms remained at the patient’s side. Both CBCT and MDCT were performed following catheterization of the RAV orifice (Fig. 1). Visipaque™ (320 mg/mL) contrast (GE Healthcare, Chicago, IL) was injected (contrast to saline ratio of 1:5) via a Nemoto Press Duo contrast injector (Siemens Healthineers, Malvern, PA) at a rate of 0.5 to 1 mL per second for a total volume of 2–3 mL. MDCT images were reconstructed in axial, coronal, and sagittal planes with 1.25 mm thickness at 1.25 mm intervals, with the axial FOV set to include all relevant anatomy. If the intraprocedural CT scan confirmed appropriate placement of the catheter within the RAV, then aspiration of the RAV was performed via a 10 mL syringe. Next, the LAV was cannulated with a Simmons 2 catheter (Cook Medical, Bloomington, IN). Finally, aspiration of 10 mL of PV blood was performed from the right common femoral vein sheath which was subsequently removed. The cohort of patients who underwent intraprocedural CT imaging during AVS were compared with the cohort of patients who did not undergo intraprocedural CT imaging to determine if there was a difference with respect to obtaining a RAV SI > 3 [6].

### Statistical analysis

Variables were summarized using median and range. Fisher’s exact tests were used for comparisons, as appropriate. Statistical analyses were performed using GraphPad (GraphPad Software, Boston, MA). *P*-values < 0.05 were considered statistically significant.

## Results

The database included 124 AVS procedures involving 110 patients performed from October 2000 through September 2022. Descriptive data for the patients and procedures are presented in Table 1.

**Table 1:** Patient characteristics

Age (median, years)	54.1 (range, 20.8–75.9)
Sex	72 M: 38 F
Body mass index (median, kg/m^2^)	32.8 (range, 20.8–57.2)
Pre-AVS blood pressure, systolic (median, range, mmHg)	149 (range, 107–184)
Pre-AVS blood pressure, diastolic (median, range mmHg)	86 (range, 61–118)
Number of pre-procedural antihypertensives (median)	3 (range, 0–7)
Plasma aldosterone concentration (median, ng/dL)	22 (range, 3–275)
CT imaging available prior to AVS (Yes/No)	118 Yes; 6 No
RAV identified in pre-AVS CT imaging (Yes, percent)	61 Yes (51.7%)
RAV SI > 3	98 (79.0%) of 124 AVS procedures

*AVS* Adrenal vein sampling, *RAV* right adrenal vein, *SI* selectivity index

Successful cannulation of the RAV, as defined as a SI > 3, was achieved in 98 (79.0%) of 124 procedures. Pre-AVS CT imaging was available prior to 118 (95.2%) of the 124 AVS procedures. On these 118 pre-AVS CT imaging datasets, the RAV was identified on 61 (51.7%) pre-AVS CT datasets. For these 61 procedures in which the RAV was identified on pre-AVS CT imaging, the RAV was successfully catheterized in 52 (85.2%) procedures and there were 9 (14.8%) unsuccessful AVS procedures. Of note, intraprocedural CT was performed during 34 (55.7%) of the 61 procedures in which pre-AVS CT imaging successfully identified the RAV. When the RAV could not be identified on pre-procedural imaging (63 procedures), the RAV was successfully catheterized in 46 (73.0%) procedures and there were 17 (27.0%) unsuccessful AVS procedures, *P* = 0.12.

There were 52 AVS procedures in which both the location of the RAV on pre-AVS CT imaging was identified and the RAV SI > 3. The mapping between the RAV location based on the pre-AVS CT images compared with the fluoroscopic images during AVS demonstrated the following:

1. 17 (32.7%) procedures showed exact concordance of the RAV location based on AVS and pre-AVS CT imaging.
2. 12 (23.1%) procedures demonstrated RAV localization 1 level cranial to the level identified on pre-AVS CT images.
3. 15 (28.8%) procedures demonstrated RAV localization 2 levels cranial to the level identified on pre-AVS CT images.
4. 7 (13.5%) procedures demonstrated RAV localization 3 levels cranial to the level identified on pre-AVS CT images.
5. 1 (1.9%) procedure demonstrated RAV localization ≥ 4 levels cranial to the level identified on pre-AVS CT images.

The rate of successful RAV cannulation during AVS was significantly greater when intra-procedural CT was performed (*n* = 60 [93.8%] of 64 procedures) compared with AVS procedures in which intra-procedural CT was not performed (*n* = 39 [63.9%] of 61 procedures) *P* < 0.01. Of note, of the 64 procedures in which intraprocedural CT was performed, 27 (42.2%) used CBCT and 37 (7.8%) used MDCT. The rate of successful RAV cannulation was 88.5% (*n* = 23 of 26 procedures) when CBCT was used during the AVS procedure compared to 97.3% (*n* = 36 of 37 procedures) when MDCT was used (*P* = 0.30). Of note, there were 33 AVS procedures in which the RAV was not identified on pre-AVS CT imaging and no intraprocedural CT was performed, and the RAV was only successfully cannulated during 17 (51.5%) of these procedures.

## Discussion

The current study compared the rate of RAV localization on pre-AVS CT imaging to the rate of RAV localization during AVS. As demonstrated on prior studies, localization of the RAV on pre-AVS CT aids the proceduralist by providing a range of potential locations for the RAV during AVS [13–16]. Interventional radiologists will typically begin searching for the RAV at T11 and then probe the IVC for 2 vertebral bodies in the cranial and caudal directions. Our study demonstrates that the range of potential RAV locations during AVS can be be further refined when its position is identified on pre-AVS CT. Specifically, we found that the RAV localized within 3 *levels* (for purposes of our study a *level* was defined as a third of a vertebral body or disk space) cranial to the location identified on pre-AVS CT imaging in 98.1% of cases. While the location of the RAV on pre-AVS CT imaging and fluoroscopy is not exact, the more limited search area to probe the IVC for the RAV orifice may facilitate decreased procedure time, radiation exposure, and contrast dose. Furthermore, we postulate that our finding that the localization of the RAV within 3 levels cranial to the location found on pre-AVS CT imaging may be secondary to the differences in respiration during pre-AVS CT imaging and AVS. Specifically, CT imaging is performed during mid-expiration compared to imaging during AVS when the patient is most often in the expiratory phase of respiration. The different pattern of respiration and the degree of sedation may account for the observed differences in localization. Another potential benefit of accurate identification of the RAV using pre-AVS CT images is to aid the proceduralist with the axis of orientation of the RAV, which may be directed in the posterior, posterolateral, or lateral positions relative to the IVC.

This study underscores the importance of pre-AVS CT imaging. While no significant difference in successful RAV cannulation during AVS were observed when the RAV orifice was identified on pre-AVS CT imaging, it is possible that these results are confounded by the utilization of intraprocedural CT in only 64 (51.6%) of 124 AVS procedures during the study period. As evidence of the importance of pre-AVS identification of the RAV on CT imaging, the rate of successful RAV catheterization in our patient cohort was low (51.5%; *n* = 17 of 33 procedures) when both of the following conditions were met: (1) pre-AVS CT imaging failed to demonstrate the RAV and (2) intraprocedural CT was not performed. The main benefit of pre-AVS identification of the RAV orifice is to pinpoint the potential area of the IVC from which the RAV originates. While not specifically assessed in this retrospective review, we surmise that limiting the range in which the RAV may originate may help to decrease fluoroscopy and procedure times during AVS.

At our institution, we utilize a specific CT imaging protocol to aid in visualization of the RAV. Prior studies have shown that the RAV is best visualized on CT using thin-slice reconstructions (0.5–0.75 mm slice thickness) and imaging during multiple phases of contrast enhancement (i.e., arterial, “late” arterial [10–15 s after the initial scan], venous [70 s after the initial scan], and delayed [3 min after the initial scan] phases) [14, 23]. The importance of a specific protocol for imaging of the adrenal glands and veins is important for two reasons: to facilitate identification of the RAV for AVS and to aid the surgeon with a priori identification of RAV aberrations (e.g., duplicated RAV or < 1 cm RAV length) that may facilitate posterior retroperitoneoscopic adrenalectomy.

**Fig. 2:**
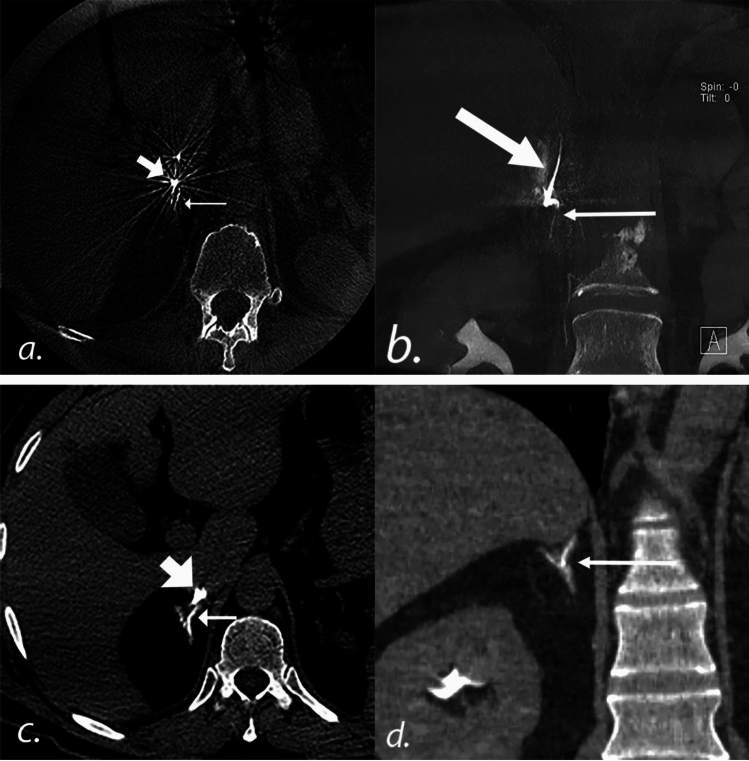
Intraprocedural CT images of the RAV during AVS. **a** 65-year-old male was referred for AVS. Axial image from an intraprocedural CBCT scan showing the RAV (*thin arrow*). Steak artifact is caused by the radiopaque marker at the tip of the C2 catheter (*thick arrow*) which is used to cannulate the RAV. **b** Coronal reconstruction demonstrates the C2 catheter (*thick arrow*) and faint contrast within the medial limb of the right adrenal gland (*thin arrow*). **c** 59-year-old male was referred for AVS. Axial image from MDCT demonstrates the RAV (*thin arrow*). While the tip of the C2 catheter (*thick arrow*) is radiopaque, the degree of streak artifact is less pronounced than on CBCT. **d** Coronal reconstruction demonstrates contrast clearly opacifying the entirety of the adrenal gland (*thin arrow*). *RAV* right adrenal vein, *AVS* adrenal venous sampling, *CBCT* cone-beam CT, *C2* Cobra-2, *MDCT* multidetector CT

This study supports previous findings of the utility of intra-procedural CT during AVS [17–21]. The rate of successful RAV sampling increased from 63.9 to 93.8% when catheter position was confirmed by intraprocedural CT acquired after contrast administration through the catheter placed into the RAV (*P* < 0.01). CBCT was used for 27 (42.2%) of 64 intraprocedural CT scans and MDCT was used in 37 (57.8%) of 64 AVS procedures. While no correlation between the type of intra-procedural CT scan used and the subsequent success of RAV catheterization was observed, there are practical differences between CBCT and MDCT. First, CBCT is limited by a relatively long acquisition time, typically 6 s, as opposed to MDCT, which had an image acquisition time of approximately 1–2 s for a comparable image volume. The long acquisition time may lead to artifacts from respiratory motion, especially if the patient is sedated as minimal or moderate sedation can impact the depth of the respiratory cycle. Second, higher available radiation output in MDCT offers flexibility in positioning of the patient’s arms. The patient’s arms were raised above their head during CBCT while the patient’s arms remained by their side during MDCT. Considering the RAV’s short length and small diameter, these arm movements during CBCT can lead to dislodgement of the catheter used to cannulate the RAV during image acquisition. Finally, the tip of the catheter used to cannulate the RAV is radiopaque to improve visualization during fluoroscopy. The radiopacity of the tip, however, causes streak artifacts in CBCT images. Streak artifacts are less severe in MDCT compared to CBCT owing to increased angular sampling and reduced beam hardening, facilitating accurate identification of the RAV during AVS (Fig. 2).

This study has the following limitations. First, this study was retrospective with its attendant biases. Operators at our institution place an emphasis on high-quality pre-AVS CT imaging as well as performing intraprocedural CT whenever the technology is available in the procedural suite. The predisposition of our practice to emphasize pre-AVS and intraprocedural CT imaging may introduce a bias towards detecting a difference. Second, the success rates of AVS procedures increased over time. The importance of physician experience for the success rate of RAV catheterization cannot be overstated. The protracted time frame of this study (22 years) introduces a confounding effect of increasing operator experience. It should be noted, however, that one of the most difficult aspects of successful RAV catheterization during AVS is the wide variation in radiographic appearance [8]. Our experience was that intra-procedural CT served to expedite the learning curve for interventional radiologists during their early experience, introducing them to the different radiographic appearances of the RAV. Third, the pre-AVS CT imaging evaluated in this study was conducted at various institutions. Prior studies have evaluated the accuracy of CT imaging in depicting the RAV. Fourth, most intraprocedural CT images were acquired using MDCT, which provides superior contrast and temporal resolution compared to CBCT. While other studies have described similar improvements with successful RAV catheterization when using CBCT, our results should be interpreted with the caveat that a MDCT imaging was used in most of the procedures in which the RAV was successfully cannulated during AVS.

Our findings corroborate the utility of pre-AVS CT imaging and intra-procedural CT imaging to facilitate successful RAV sampling during AVS. Given the high prevalence of hypertension and the suspected underdiagnosis of PA, utilization of AVS will likely increase. Success of the procedure is primarily contingent upon cannulation of the RAV. This work also provides a CT-guided roadmap for improving the success of AVS.

## References

[1] Douma S, Petidis K, Doumas M, Papaefthimiou P, Triantafyllou A, Kartali N, et al. Prevalence of primary hyperaldosteronism in resistant hypertension: a retrospective observational study. Lancet. 2008;371(9628):1921-6. 18539224 10.1016/S0140-6736(08)60834-X

[2] Kayser SC, Dekkers T, Groenewoud HJ, van der Wilt GJ, Carel Bakx J, van der Wel MC, et al. Study Heterogeneity and Estimation of Prevalence of Primary Aldosteronism: A Systematic Review and Meta-Regression Analysis. J Clin Endocrinol Metab. 2016;101(7):2826-35. 27172433 10.1210/jc.2016-1472

[3] Young WF. Primary aldosteronism: renaissance of a syndrome. Clin Endocrinol (Oxf). 2007;66(5):607-18. 17492946 10.1111/j.1365-2265.2007.02775.x

[4] Funder JW, Carey RM, Mantero F, Murad MH, Reincke M, Shibata H, et al. The Management of Primary Aldosteronism: Case Detection, Diagnosis, and Treatment: An Endocrine Society Clinical Practice Guideline. J Clin Endocrinol Metab. 2016;101(5):1889-916. 26934393 10.1210/jc.2015-4061

[5] Doppman JL, Gill JR, Jr. Hyperaldosteronism: sampling the adrenal veins. Radiology. 1996;198(2):309-12. 8596821 10.1148/radiology.198.2.8596821

[6] Rossi GP, Auchus RJ, Brown M, Lenders JW, Naruse M, Plouin PF, et al. An expert consensus statement on use of adrenal vein sampling for the subtyping of primary aldosteronism. Hypertension. 2014;63(1):151-60. 24218436 10.1161/HYPERTENSIONAHA.113.02097

[7] Sam D, Kline GA, So B, Leung AA. Discordance Between Imaging and Adrenal Vein Sampling in Primary Aldosteronism Irrespective of Interpretation Criteria. J Clin Endocrinol Metab. 2019;104(6):1900-6. 30590677 10.1210/jc.2018-02089

[8] Daunt N. Adrenal vein sampling: how to make it quick, easy, and successful. Radiographics. 2005;25 Suppl 1:S143-58. 16227488 10.1148/rg.25si055514

[9] Georgiades CS, Hong K, Geschwind JF, Liddell R, Syed L, Kharlip J, et al. Adjunctive use of C-arm CT may eliminate technical failure in adrenal vein sampling. J Vasc Interv Radiol. 2007;18(9):1102-5. 17804771 10.1016/j.jvir.2007.06.018

[10] Kinnison M. Adrenal vein sampling with C-arm CT. J Vasc Interv Radiol. 2008;19(1):153; author reply 18192484 10.1016/j.jvir.2007.10.004

[11] Yoshida K, Kobayashi S, Matsui O, Gabata T, Sanada J, Koda W, et al. Hepatic pseudolymphoma: imaging-pathologic correlation with special reference to hemodynamic analysis. Abdom Imaging. 2013;38(6):1277-85. 23744440 10.1007/s00261-013-0016-6

[12] Miotto D, De Toni R, Pitter G, Seccia TM, Motta R, Vincenzi M, et al. Impact of accessory hepatic veins on adrenal vein sampling for identification of surgically curable primary aldosteronism. Hypertension. 2009;54(4):885-9. 19687347 10.1161/HYPERTENSIONAHA.109.134759

[13] Degenhart C, Strube H, Betz MJ, Pallauf A, Bidlingmaier M, Fischer E, et al. CT mapping of the vertebral level of right adrenal vein. Diagn Interv Radiol. 2015;21(1):60-6. 25430527 10.5152/dir.2014.14026 PMC4463352

[14] Omura K, Ota H, Takahashi Y, Matsuura T, Seiji K, Arai Y, et al. Anatomical Variations of the Right Adrenal Vein: Concordance Between Multidetector Computed Tomography and Catheter Venography. Hypertension. 2017;69(3):428-34. 28137990 10.1161/HYPERTENSIONAHA.116.08375

[15] He X, Sueyoshi E, Tasaki Y, Miyazaki S, Murakami T, Nagayama H, et al. Benefits of adrenal venous sampling with preoperative four-dimensional CT imaging. Acta Radiol. 2023;64(3):1280-9. 35945822 10.1177/02841851221118799

[16] Morita S, Nishina Y, Yamazaki H, Sonoyama Y, Ichihara A, Sakai S. Dual adrenal venous phase contrast-enhanced MDCT for visualization of right adrenal veins in patients with primary aldosteronism. Eur Radiol. 2016;26(7):2073-7. 26494644 10.1007/s00330-015-4073-9

[17] Kunkel ME, Herkommer A, Reinehr M, Bockers TM, Wilke HJ. Morphometric analysis of the relationships between intervertebral disc and vertebral body heights: an anatomical and radiographic study of the human thoracic spine. J Anat. 2011;219(3):375-87. 21615399 10.1111/j.1469-7580.2011.01397.x PMC3171774

[18] 17.Onozawa S, Murata S, Tajima H, Yamaguchi H, Mine T, Ishizaki A, et al. Evaluation of right adrenal vein cannulation by computed tomography angiography in 140 consecutive patients undergoing adrenal venous sampling. Eur J Endocrinol. 2014;170(4):601-8. 24459237 10.1530/EJE-13-0741

[19] 18.Maruyama K, Sofue K, Okada T, Koide Y, Ueshima E, Iguchi G, et al. Advantages of Intraprocedural Unenhanced CT During Adrenal Venous Sampling to Confirm Accurate Catheterization of the Right Adrenal Vein. Cardiovasc Intervent Radiol. 2019;42(4):542-51. 30519725 10.1007/s00270-018-2135-5

[20] 19.Meyrignac O, Arcis E, Delchier MC, Mokrane FZ, Darcourt J, Rousseau H, et al. Impact of cone beam - CT on adrenal vein sampling in primary aldosteronism. Eur J Radiol. 2020;124:108792. 31926384 10.1016/j.ejrad.2019.108792

[21] 20.Hafezi-Nejad N, Gullotti DM, Bailey CR, Lessne ML, Holly BP. Does Intraprocedural CT Improve the Success Rate of Adrenal Venous Sampling? A Systematic Review and Meta-Analysis of Data from 809 Patients. Cardiovasc Intervent Radiol. 2022;45(1):29-40. 34518912 10.1007/s00270-021-02954-7

[22] 21.Siragy HM, Vieweg WV, Pincus S, Veldhuis JD. Increased disorderliness and amplified basal and pulsatile aldosterone secretion in patients with primary aldosteronism. J Clin Endocrinol Metab. 1995,80(1):28-33. 7829626 10.1210/jcem.80.1.7829626

[23] 22.Matsuura T, Takase K, Ota H, Yamada T, Sato A, Satoh F, et al. Radiologic anatomy of the right adrenal vein: preliminary experience with MDCT. AJR Am J Roentgenol. 2008;191(2):402-8. 18647909 10.2214/AJR.07.3338

